# Genome-Wide Characterization and Expression Analysis of Pathogenesis-Related 1 (*PR-1*) Gene Family in Tea Plant (*Camellia sinensis* (L.) O. Kuntze) in Response to Blister-Blight Disease Stress

**DOI:** 10.3390/ijms23031292

**Published:** 2022-01-24

**Authors:** Qiqi Zhang, Nini Guo, Yongheng Zhang, Youben Yu, Shuyuan Liu

**Affiliations:** College of Horticulture, Northwestern Agricultural and Forestry University, Xianyang 712000, China; zqqzqq@nwafu.edu.cn (Q.Z.); gnn@nwafu.edu.cn (N.G.); zhangyongheng@nwafu.edu.cn (Y.Z.); chyyjs@nwsuaf.edu.cn (Y.Y.)

**Keywords:** PR-1, blister blight, *Camellia sinensis*, defense mechanism

## Abstract

Pathogenesis-related 1 (PR-1) proteins, which are defense proteins in plant–pathogen interactions, play an important role in the resistance and defense of plants against diseases. Blister blight disease is caused by *Exobasidium vexans* Massee and a major leaf disease of tea plants (*Camellia sinensis* (L.) O. Kuntze). However, the systematic characterization and analysis of the *PR-1* gene family in tea plants is still lacking, and the defense mechanism of this family remains unknown. In this study, 17 *CsPR-1* genes were identified from the tea plant genome and classified into five groups based on their signal peptide, isoelectric point, and C-terminus extension. Most of the CsPR-1 proteins contained an N-terminal signal peptide and a conserved PR-1 like domain. *CsPR-1* genes comprised multiple *cis*-acting elements and were closely related to the signal-transduction pathways involving TCA, NPR1, EDS16, BGL2, PR4, and HCHIB. These characteristics imply an important role of the genes in the defense of the tea plant. In addition, the RNA-seq data and real-time PCR analysis demonstrated that the *CsPR-1-2*, -4, *-6*, *-7*, *-8*, *-9*, *-10*, *-14*, *-15,* and *-17* genes were significantly upregulated under tea blister-blight stress. This study could help to increase understanding of *CsPR-1* genes and their defense mechanism in response to tea blister blight.

## 1. Introduction

The plant immune system has multiple layers of defense responses to intercept the infection and damage caused by pathogenic microorganisms [1]. In the first barrier of the immune system, plants recognize the conserved pathogen-associated molecular patterns (PAMPs) of slowly evolving microorganisms or pathogens through the pattern-recognition receptors (PRRS) located on the surface of the cell membrane to activate pattern-triggered immunity (PTI) [2]. However, compatible pathogens can overcome this first layer of defense. Plants specifically recognize effectors via polymorphic nucleotide-binding-domain and leucine-rich repeat (NB-LRR) proteins encoded by most resistance genes (*R* genes) directly or indirectly to activate effector-triggered immunity (ETI) [3]. According to the products encoded by defense genes and their functions, these genes can be divided into secondary metabolite-synthesis genes, cell-wall-modification -related genes, protease-inhibitor genes, and plant-pathogenesis-related (*PR*) genes [4].

Numerous studies have shown that *PR* genes play important roles in the resistance and defense of plants against diseases [5,6,7]. PR proteins are induced and accumulated in host plants during attacks by oomycetes, fungi, bacteria, viruses, or insects. Seventeen families of PR proteins have been classified and characterized according to the homology of amino-acid sequences, serological relationships, and enzyme activities [8]. PR-1 proteins are related to cysteine-rich secretory proteins, the core regions of which are relatively conservative in various species [9]. The existence of a PR-1-like domain helps to deal with the stress of an adverse environment and improves the stability of PR-1-like proteins [10]. The CAP-derived peptide 1 (CAPE1, consensus motif PxGNxxxxxPY) has been found in the PR-1-like domain, which is related to stress signaling and conserved between the monocots and dicots [11]. PR-1 proteins occur in multigene families within plant genomes and can be further classified as acidic or basic, depending on their theoretical isoelectric point (p*I*) [12]. Numerous studies have demonstrated that PR-1 proteins abundantly accumulate during plant-defense responses and are ubiquitous across plant species [8,13]. In grapevine (‘BN5-4’), the basic-type *VvPR1b1* gene confers high resistance against *Pseudomonas syringae* [14]. The WjAMP-2 (PR-1) protein has been purified from *Wasabia japonica* and strongly inhibits activity against phytopathogenic fungi, and the overexpression of *WjAMP-2* in tobacco could enhance resistance to *Botrytis cinerea* [15]. In addition, *PR-1* gene expression has been considered a reliable marker for the salicylic-acid (SA)-mediated defense pathway and the activation of systemic acquired resistance (SAR) in various plant species [16]. Recently, with the development of gene-sequencing technology and the improvement of bioinformatics prediction systems, a growing number of gene families has been identified [17,18,19,20,21,22]. At present, the *PR-1* gene family has been identified, as well expression profiles in response to biotic and abiotic stresses in many species—for instance, 11 genes in *Piper nigrum* [13], 13 in *Solanum lycopersicum* [23], 21 in *Vitis vinifera* [14], 22 in *Arabidopsis* [8], 23 in *Triticum aestivum* [24], 24 in *Glycine max* [25], and 32 in *Oryza sativa* [8]. Clarifying the classification and composition of gene-family members in the genome is the first step to explore species-related characteristics and biological problems and can lay a solid foundation for subsequent gene-function characterization and genetic manipulation.

Tea, which is the most consumed nonalcoholic beverage worldwide, is made of the apical buds and tender leaves of tea plants (*Camellia sinensis* (L.) O. Kuntze) [26]. During the growth of these plants, tea leaves are susceptible to various fungal pathogens, severely reducing the yield and quality of tea. Tea blister blight disease, caused by *Exobasidium vexans* Massee, is one of the serious diseases of tea plants [27]. *E. vexans* mainly damages the tender leaves and stems of tea plants, causing the young, diseased tissues to be covered with white blisters [28]. As the disease develops, the fungal infection leads to necrosis and withering of a large number of leaf tissues, and the tea leaves gradually turn brown and curl [29]. Nevertheless, the resistance mechanism of the response of tea plants to blister blight disease has not yet been explained in detail. Transcriptome analysis has identified 149 putative key defense-related transcripts/genes involving defense-related enzymes, resistance genes, multidrug-resistant transporters, and transcription factors [30]. Foliar application of 0.01% chitosan could induce the expression of different defense-related enzymes via NO signaling to reduce the incidence of blister blight [31]. Although an important gene family in the resistance and defense of plants against diseases, *PR-1* genes have not been revealed in tea plants, and the biological functions of the members of this family remain unknown. In this study, *CsPR-1* genes were identified based on transcriptome data analysis and the tea genome database. The genes were characterized, and expression patterns were detected and analyzed. The results could provide an important foundation for the exploration of the biological functions of the *CsPR-1* gene family in tea plants.

## 2. Results

### 2.1. Identification and Characterization of CsPR-1 Gene-Family Members

Using the Tea Plant Genome Database and bioinformatics analysis, 17 *CsPR-1* gene members and their corresponding amino-acid sequences were confirmed and listed in Table S1. These *CsPR-1* genes were named *CsPR-1-1* to *CsPR-1-17* according to the sequence of their genomic output. The CsPR-1 proteins ranged from 156 aa (CsPR-1-10) to 340 aa (CsPR-1-2), and the molecular weights (MWs) were between 17.37 kDa (CsPR-1-10) and 38.28 kDa (CsPR-1-2). The p*I* values varied from 4.84 (CsPR-1-10) to 10.07 (CsPR-1-2), indicating the presence of PR-1 protein-encoding genes with both acidic and basic amino acids in the tea plant genome. Hydropathicity (GRAVY) analysis showed that the GRAVY values of all analyzed *CsPR-1* genes were negative, indicating their high hydrophilicity. More detailed information, including the sequence ID, chromosomes, and subcellular localization, is listed in Table 1.

### 2.2. Analysis of the Genomic and Domain Structures of CsPR-1 Genes

**Figure 1:**
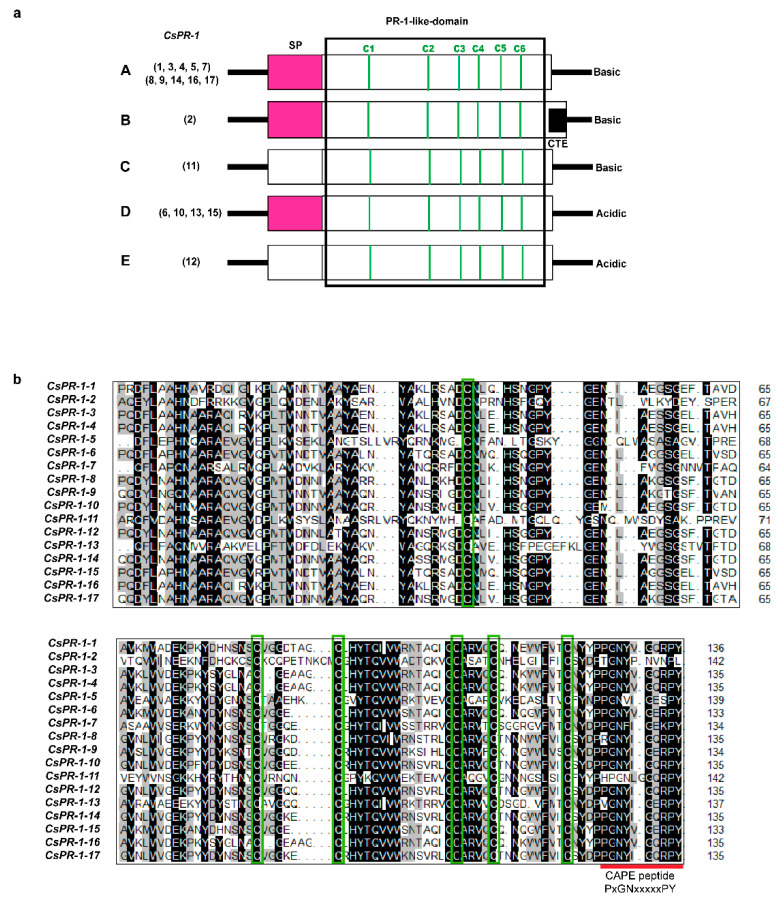
Genomic and domain structures of the *CsPR-1* genes. (**a**) Diagram of the genomic structures of the *CsPR-1* genes. Open boxes signify the open reading frames (ORFs). Signal-peptide (SP) regions are shaded in pinkish red. Vertical green solid bars represent the positions of the six conserved cysteine residues (C1-C6). The box drawn with black solid lines indicates the conserved PR-1-like domains (pfam cd05381). The interior black box indicates the C-terminal extension (CTE). (**b**) Amino-acid alignment of the PR-1-like domain of the deduced CsPR-1 proteins. DNAMAN 7.0 was used to mark the amino-acid residues with an identity of more than 50% with black shade. The areas in the interior green boxes and the red solid line indicate the C1-C6 and CAPE peptide, respectively.

According to the calculated p*I* values, the predicted signal peptide (SP), and the C-terminal extension (CTE) of the deduced proteins, the 17 *CsPR-1* genes were classified into five major groups (Figure 1a). Group A included 10 genes (*CsPR-1-1*, *-3*, *-4*, *-5*, *-7*, *-8, -9*, *-14*, *-16,* and *-17*), which all encoded basic (p*I* = 7.08-9.17) PR-1 proteins containing an N-terminal SP and a PR-1 like domain of 134 aa to 139 aa. Group B included only *CsPR-1-2*, which encoded basic (p*I* = 10.64) protein containing an SP and a PR-1-like domain of 142 aa, followed by a CTE. Group C was comprised of *CsPR-1-11*, encoded basic (p*I* = 9.13) protein with a PR-1-like domain of 141 aa. Group D included four genes (*CsPR-1-6*, *-10, -13, -14,* and *-15*), which all encoded basic (p*I* = 4.75–6.49) proteins containing an SP, followed by a conserved PR-1like domain of 133 aa to 137 aa. Group E (*CsPR-1-12*) encoded a basic (p*I* = 5.07) protein containing a PR-1 like domain of 135 aa. The amino-acid sequences of the PR-1-like domain of the 17 deduced CsPR-1 proteins were aligned. Most CsPR-1 proteins have three strictly conserved cysteine-residue pairs and a CAPE 1 peptide (Figure 1b).

### 2.3. Conserved Motifs of the CsPR-1 Protein Structure

**Figure 2:**
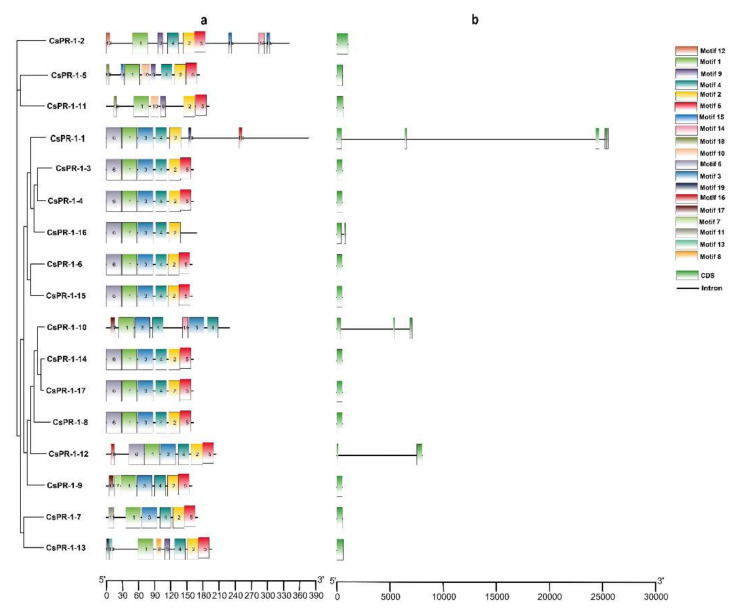
Phylogenetic clustering, motif compositions, and gene structures of the *CsPR-1* family members in tea plants. (**a**) The motifs of each CsPR-1, as well as 20 different motifs, are represented by different colored boxes. (**b**) Exon-intron structural analyses of *CsPR-1*.

To explore the structural diversity of the *CsPR-1* genes in tea plants, 20 conserved motifs in the *CsPR-1* gene family were predicted by multiple-expectation maximization for motif elicitation (MEME) (Table S2). All CsPR-1 proteins contained motifs 1 and 2. Motif 3 was present in all CsPR-1 proteins, except in CsPR-1-2, -5, -11, and -13. Motif 4 was present in all CsPR-1 proteins, except in CsPR-1-11. Motif 5 was present in all CsPR-1 proteins, except in CsPR-1-1, -10, and -16. Motifs 7, 8, and 12 were present, along with CsPR-1-9, -13, and -2, respectively (Figure 2a). Exon-intron diagrams of the *CsPR-1* genes were further generated based on their genome sequences and corresponding coding sequences. Most of the tea plant *CsPR-1* genes did not contain introns, except for *CsPR-1-1*, *-10*, *-12,* and*-16*, which contained between one and three introns (Figure 2b). A visualization of the conserved motif and gene structure clearly shows that most members of the tea plant *CsPR-1* gene family have similar structures.

### 2.4. Functional Interaction Networks of CsPR-1 Proteins

**Figure 3:**
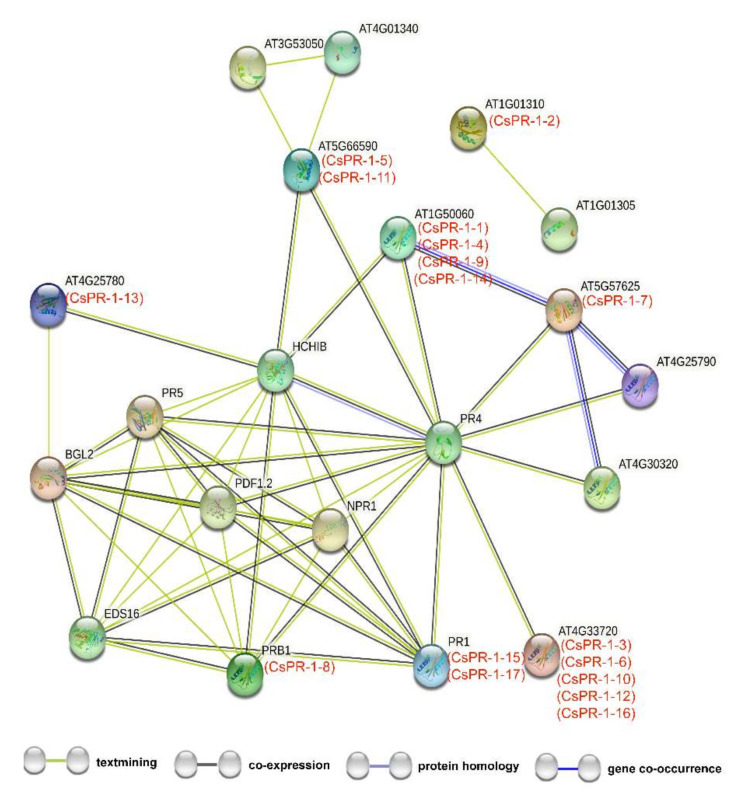
Functional interaction networks of the CsPR-1 protein in the tea plant according to orthologs in *Arabidopsis* are shown in red and black.

Functional interaction networks of the CsPR-1 proteins were constructed using STRING software on the basis of the homologous proteins in *Arabidopsis* (Table S3). The results show that the 17 CsPR-1 proteins participated in the interaction network, indicating a universal and complex interaction of CsPR-1 proteins (Figure 3). Most CsPR-1 proteins were involved in the SA-signaling pathway or JA/ET-signaling pathway by interacting with NPR1, EDS16, BGL2, PR4, and HCHIB. CsPR-1-12 was independent.

### 2.5. Analysis of Cis Elements of the CsPR-1 Genes

**Figure 4:**
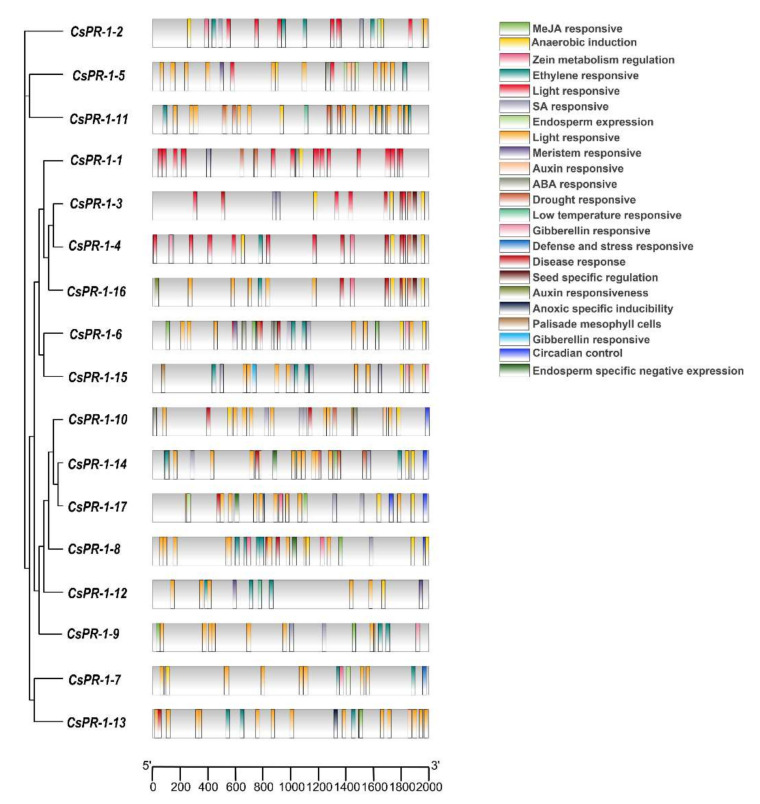
Schematic model of the cis elements of the *CsPR-1* promoter regions generated by TBtool software.

The *cis* element in the *CsPR-1* promoters were analyzed using Plant CARE. The results show many hormone-response elements, biological stress-response elements, and abiotic stress-response elements in the *CsPR-1* promoters (Figure 4). Various response-hormone elements, such as SA (TCA-element, as-1), Me-JA (TGACG-motif, CGTCA-motif), ABA (ABRE), GA (P-box, GARE-motif), ET (ERE), and IAA (TGA-element, AuxRR-core) were present. These hormones, especially SA and JA, are closely related to the resistance. SA and JA *cis* elements were present in the promoters of *CsPR-1-1, -2*, *-3*, *-4*, *-5*, *-6*, *-7*, *-8*, *-9*, *-10*, *-13*, *-14*, *-15*, and *-17* genes, and JA *cis* elements were present in *CsPR-1-2*, *-5*, *-6*, *-8*, *-9*, *-10*, *-13*, *-14*, *-15*, and *-17*. Flavonoid biosynthesis (MBSI) and W-box elements were also found in the deduced *CsPR-1* promoter, closely related to the response to fungal stress. In addition, *cis* -acting elements, in response to light (i.e., I-box, Box 4, G-box, GT1-motif, Box II, TCT motif, and L-box), low temperature (LTR), drought (MBS), anaerobic stress (ARE), and meristem (CAT-box) also exist in the promoter region of *CsPR-1* family genes.

### 2.6. Predicted Secondary and 3D Structures of CsPR-1 Proteins

**Figure 5:**
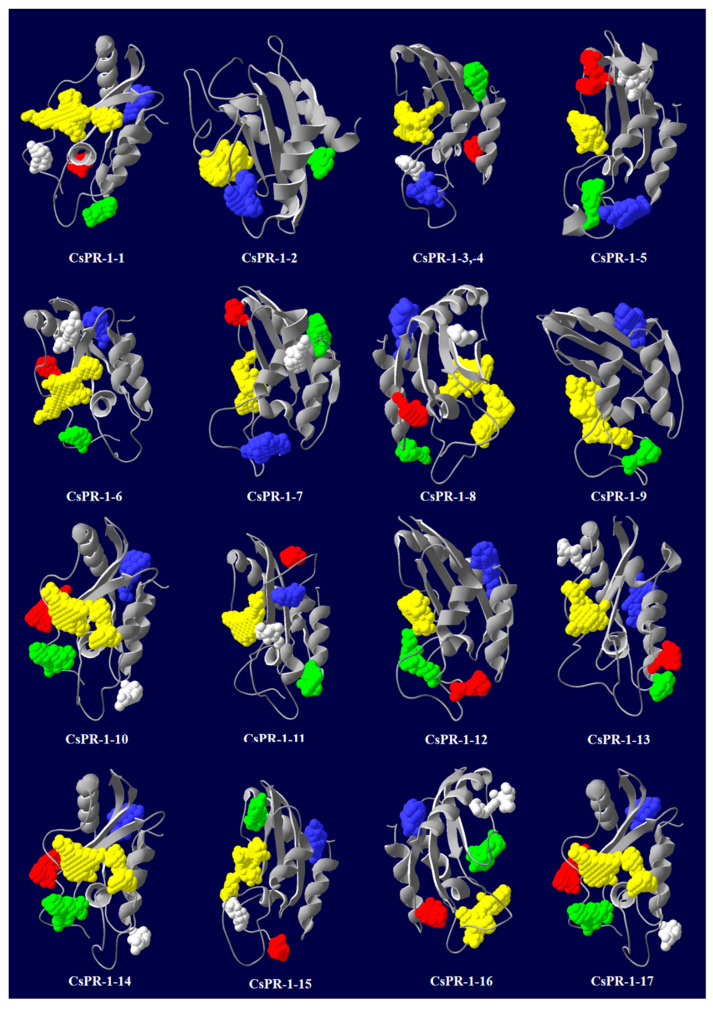
Predicted 3D structure of the CsPR-1 proteins using the Phyre2 server and pockets using the Pasaccca server. The top five predicted pockets are indicated as yellow, blue, green, red, and grey, respectively.

Secondary structures were analyzed using the SOPMA online server. The results show that α-helices, extended strands, beta turns, and random coils were found in the ranges of 23.82–39.88%, 8.24–22.45%, 1.73–7.05%, and 39.74–50.29%, respectively (Table 2). The 3D structures of the CsPR-1 proteins were predicted using the Phyre2 online server. The Ramachandran plot representing the residues in core, allowed, and generous regions exceeded 95%, revealing the quality and reliability of the protein structures (Table 2). The predicted channel structures of CsPR-1 proteins ranged from 2 to 9, and the overall percentages of disordered regions were between 6.47% and 61.76% (Table 2). The 3D structures of the CsPR-1 proteins were conserved, except for that of CsPR-1-2. The 3D structures and predicted pockets are shown in Figure 5.

### 2.7. Histomorphological Observation of Tea Leaves

**Figure 6:**
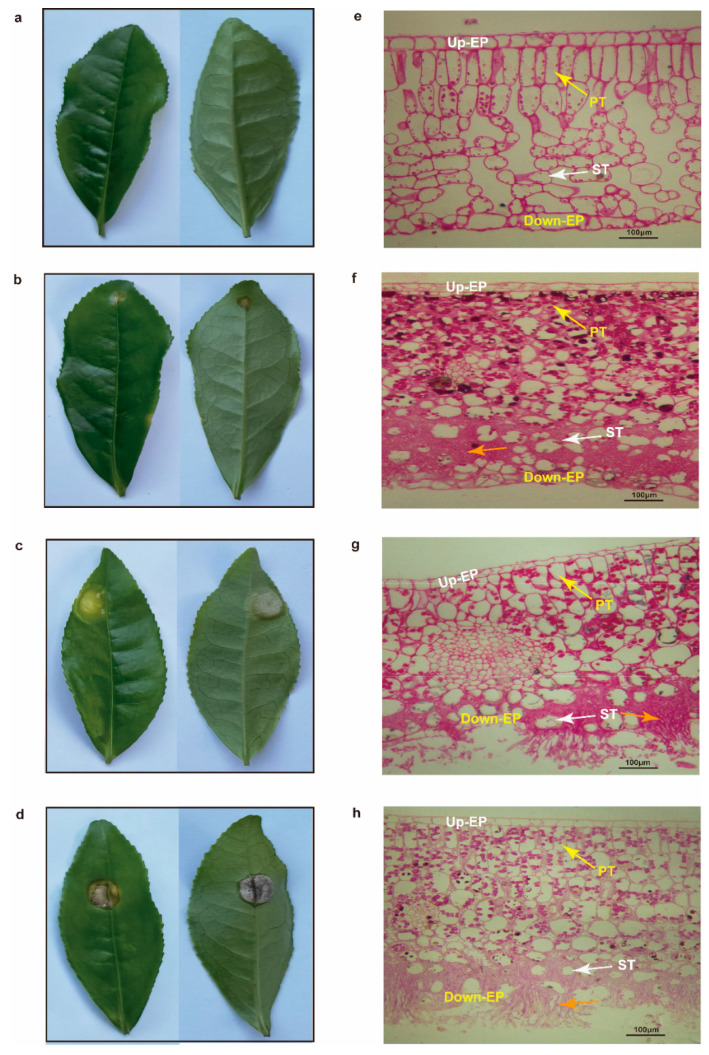
Observation of the stages and PAS staining of tea leaves. Healthy leaves (**a**,**e**) and tea leaves infected with tea blister blight at the different stages of infection: early stage (**b**,**f**), middle stage (**c**,**g**), and late stage (**d**,**h**). The orange arrow represents club-shaped hyphae structures of *Exobasidium vexans*. Up-EP: up-epidermis; Down-EP: down-epidermis; PT: palisade tissue; ST: spongy tissue. Bar = 100 μm.

Healthy tea leaves had a complete cell structure, including cuticle, upper epidermis, palisade tissue, sponge tissue, down epidermis, and cuticle, and the glycogen and neutral mucus in the palisade tissue cells were red (Figure 6a,e). However, the cell structure of leaves invaded by tea blister blight disease was infected to varying degrees. When the pathogen of tea blister blight disease invaded in the early stage, an obvious hyphal invasion was observed in the gap of the tea plant in down-epidermal cells, but the down-epidermal cells were still intact (Figure 6b,f). In the middle stage of pathogen invasion, the hyphae proliferated rapidly in the gap between tea cells. Some down-epidermal cells of the tea leaves were broken by hyphae, and a small amount of basidiospores broke through the stratum corneum in clusters (Figure 6c,g). In the late stage of pathogen invasion, the hyphae occupied and destroyed most of the sponge-tissue cells, and a large number of basidiospores grew outward in clusters, while the down-epidermal cells of the tea leaves were almost invisible (Figure 6d,h).

### 2.8. Expression Patterns of CsPR-1 Genes

**Figure 7:**
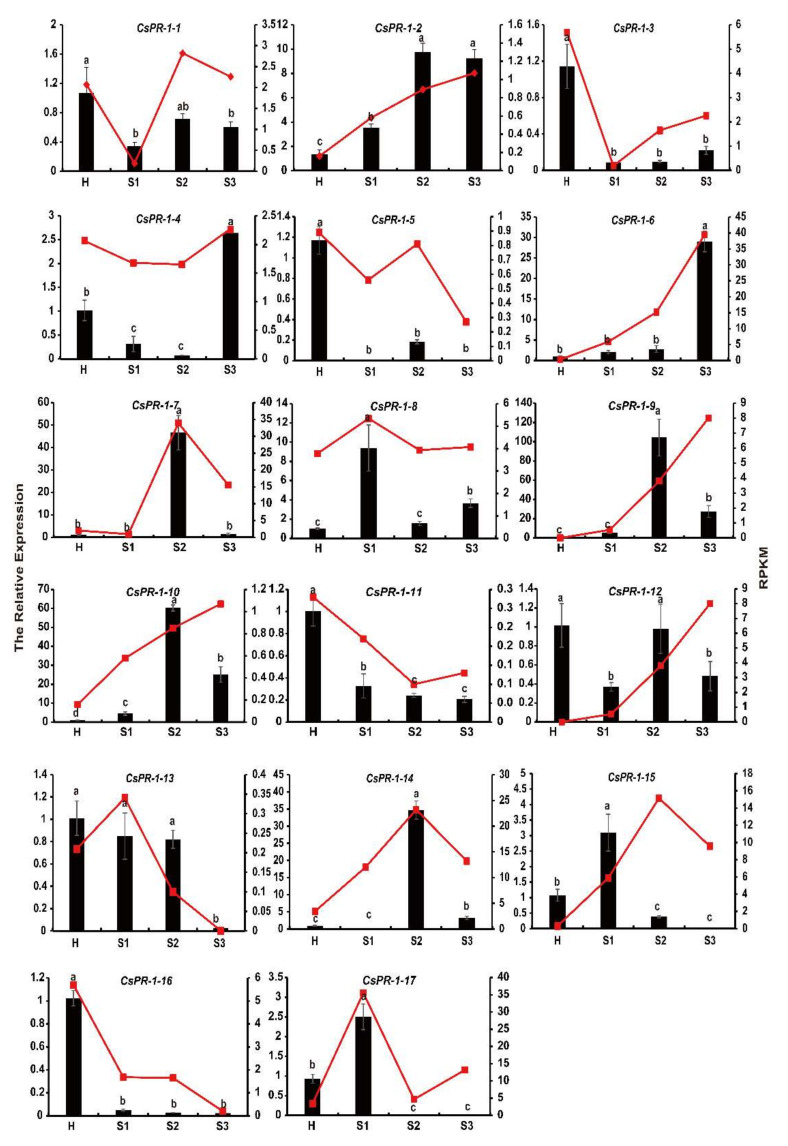
Comparison of the transcript expression results from the RNA-seq and qRT-PCR analysis of the *CsPR-1* family genes under tea blister-blight disease stress. The relative expression of genes validated using qRT-PCR (bar chart, left ordinate) and the fragments per kilobase of transcript per million mapped reads obtained from transcriptome sequencing (line diagram, right ordinate).

To further investigate the response of *CsPR-1* genes to blister blight disease and to verify the accuracy of the RNA-seq data, the expression of 17 *CsPR-1* genes in uninoculated (healthy) and inoculated (early, middle, and late stages) *E. vexans* was measured by qRT-PCR. The results show that the change trend of the expression pattern of most *CsPR-1* genes after *E. vexans* invasion in tea plants was basically similar to that of transcriptome sequencing (Figure 7). A strong response of the *CsPR-1* genes was observed, in which 10 *CsPR-1* genes (*CsPR-1-2*, -4, *-6*, *-7*, *-8*, *-9*, *-10*, *-14*, *-15,* and *-17*) were obviously upregulated (corresponding to 6.86, 2.59, 28.78, 40.37, 9.36, 97.59, 25.84, 36.85, 2.87, and 2.67 folds), whereas 7 *CsPR-1* genes (*CsPR-1-1*, *-3*, *-5*, -11, -12, *-13,* and *-16*) were significantly downregulated (corresponding to 0.32, 0.07, 0.15, 0.20, 0.36, 0.02, and 0.02 folds). The expression levels of these genes showed significant differences across the different disease stages. *CsPR-1-2*, *-7*, -8, *-9*, *-10*, *-14*, *-15,* and *-17* expression was significantly increased during the early and middle stages, while the expression of *CsPR-1-4* and *-6* was upregulated in the late stage.

## 3. Discussion

PR-1 is a kind of PR protein induced by the host against a pathogen and encoded by multigene families in plants [8]. Seventeen members of the *PR-1* gene family from the tea genome were identified in the current study, consistent with similar investigations in other plant species. Comprehensive analyses of the gene structures, conserved motifs, *cis* elements, protein-interaction networks, and expression patterns were performed, and the results could provide a scientific basis for future functional research.

The PR-1 family is highly conserved and exists in all plants studied. This family has been confirmed to possess an antifungal function. According to p*I* values, PR-1 proteins can be broadly classified as either acidic or basic proteins [12]. These two protein types have different inhibitory activities against fungal pathogens. In tomato plants, basic PR-1c and PR-1g exhibit greater antifungal activity against *Phytophthora infestans* than acidic PR-1a and PR-1b [32]. Overexpression of TMV-inducible basic pepper *PR-1* gene in tobacco leaves could significantly enhance the resistance to heavy-metal stresses and pathogens [33]. In the current study, most basic CsPR-1 proteins were found to be more rapidly and strongly expressed than acidic CsPR-1 proteins in tea plants during interaction with *E. vexans*. This finding is similar to those reported by previous studies. The basic *CsPR-1-2*, *-4*, *-7*, *-8*, *-9**,** -14*, and *-17* genes were obviously upregulated, and most of these genes responded rapidly during the early or middle stages of infection. These characteristics indicate that these genes may play an important role in the early pathogenesis of tea blister blight disease. Acidic *CsPR-1-6**,** -10*, and *-15* were also upregulated. The expression trends of *CsPR-1* genes were diverse, indicating a complex defense mechanism to tea blister blight in tea plants.

Notably, all deduced amino-acid sequences of *CsPR-1* genes, except for those of *CsPR-1-11* and*-12*, contained an SP at the N terminal. An SP is an RNA region encoding hydrophobic amino-acid sequences, which, in most cases, is a transient extension to the amino terminus of the protein and is responsible for guiding proteins into subcellular organelles with different membrane structures [34]. Previous studies on *Triticum aestivum* and *Vitis* showed that all PR-1 proteins contain an SP at the N terminal, which could secrete PR-1 proteins into the extracellular environment [14,24]. Two PnPR-1 proteins without SPs were also found in *Piper nigrum*, which is consistent with the present results [13]. Most CsPR-1 proteins containing an SP at the N terminal might be guided into special subcellular organelles for their biological functions. The CTE is supposed to be functionally similar to the carboxyl-terminal propeptide encoded by *PR-2* and *PR-3* genes, which is known to serve as a vacuolar targeting signal [16,35]. The CTE domain has been found in both dicot and monocot species, such as tobacco *PR-1b* and tomato *PR-1a1* [36,37]. In this study, only CsPR-1-2 was found to contain the CTE domain, which may play an important role in guiding CsPR-1 proteins into vacuolar targeting. The 3D structures of CsPR-1 proteins are also conserved, except for those of CsPR-1-2. The structural variation was significantly connected with the CTE domain of the CsPR-1-2 gene and resulted in different function of PR-1 proteins in tea plants. Disordered regions contain flexibility-intrinsic conformation to bind multiple partners in transient interactions and are closely related to transcriptional regulation, signaling cascade, cell-cycle control, and chaperone activity [38]. The presence of disordered regions in CsPR-1-2 was up to 61.76%, which might be related to the metabolic roles of these proteins in cellular regulation. Further characterization may provide a theoretical basis for the exploration of the structural and functional diversity of CsPR-1 proteins in tea plants.

Previous studies have shown that the multigene family was formed by duplication and mutation of an ancestral gene [39]. The survival of duplicate genes made the regulatory and coding regions tend to differentiate, leading to differences in the structure and function of genes in the same family [39]. In the current study, the most closely related *CsPR-1* genes in the same group were found to share a similar conserved motif and gene structures, indicating that analysis of the motif and gene structures was contributed to understanding of the evolutionary relationships of *CsPR-1* family genes. Furthermore, introns are less conserved compared with exons. Intron density tended to decline during evolution in some genes that need to rapidly activate in response to stress, such as heat-shock proteins [40]. Using molecular characterization and sequence analysis, investigators in a previous study found that *PR-1* genes lack introns [41]. In the current investigation, a majority of *CsPR-1* genes were found to have no introns or only between one and three introns, which was conducive to the transcriptional regulation of these genes under stress. *CsPR-1-7*, *-9*, and *-14* without introns in the same group responded more rapidly to tea blister blight disease stress than *CsPR-1-1*, which contains three introns.

Although substantial evidence has demonstrated that PR-1 acts as a defense protein in plant-pathogen interactions, the biological functions of PR-1 proteins remain obscure. A previous study showed that SA and JA can induce the expression of up- and downregulated *PR* genes, respectively [42]. *PR-1* gene in wheat was strongly induced after ABA and SA treatments [43]. Similarly, under cold treatment and exogenous application of SA, the expression of *PR-1* genes in *Arabidopsis* was significantly upregulated [44]. In the interaction networks of *CsPR-1*, many key genes engaged in activating and mediating diverse defense responses were involved. AT5G66590 (CsPR-1-5 and -11), AT5G57625 (CsPR-1-7), and AT4G33720 (CsPR-1-3, -6, -10, and -12) interacted with PR4, which was highly related to the defense of grapevine against downy mildew resistance and related to the JA/ET-signaling pathway [45]. HCHIB was basic endochitinase B, related to the JA/ET-mediated signaling pathway during systemic acquired resistance, which is an enzyme known for antifungal activity and close interaction with AT1G50060 (CsPR-1-1, -4, -9, and -14) and AT4G25780 (CsPR-1-13) [40]. CsPR-1-15 and -17 were involved in the defense of SA response. NPR1 was found to positively modulate SA signaling in plants and to interact with PRB1 (CsPR-1-8) [46]. CsPR-1 proteins were found to be involved in diverse disease resistance and to play a connecting role in the complex response network of different signal pathways.

## 4. Materials and Methods

### 4.1. Plant Materials

Tea plants (*Camellia sinensis cv.* Fuding dabai) were grown in the gardens of Northwest A&F University Tea Experimental Station (Xixiang, Shaanxi, China, 32°57′43″ N, 107°40′12″ E). Tea leaves (from the third leaf from the top of the plant) with typical tea blister-blight symptoms were sampled within 4 h. Each tea leaf only had one blister. The severity was classified into three grades on the basis of the course of the disease, as previously reported: early stage (S1, the formation of yellow, transparent patches with a diameter of 2–4 mm), middle stage (S2, the formation of features blisters), and late stage (S3, the formation of necrotic spots) [47]. Healthy tea leaves (H) free of any infestation at the same leaf positions were used as control. All samples were immediately frozen in liquid nitrogen and stored at −80 °C for periodic acid-Schiff (PAS) staining, RNA-seq, and real-time PCR (qRT-PCR) with three biological replicates.

### 4.2. Database Mining and Identification of CsPR-1 Genes

Published *Arabidopsis PR-1* sequences were used as queries for BLASTP searches against the Tea Plant Genome Database (http://tpia.teaplant.org/index.html, accessed on 16 September 2021) [48]. All output genes were then verified using Pfam (http://pfam.xfam.org/family/PF00188, accessed on 16 September 2021). SMARAT (http://smart.embl-heidelberg.de/, accessed on 16 September 2021) and CCD (https://www.ncbi.nlm.nih.gov/cdd/, accessed on 16 September 2021) were used to confirm all the putative *CsPR-1* genes. The chromosomal localization information of all *CsPR-1* genes was obtained by local blast of TPIA (http://tpia.teaplant.org/index.html, accessed on 17 September 2021). p*I* and molecular weight (MW) were predicted using ProtParam (http://web.expasy.org/protpara-m/, accessed on 17 September 2021). The online program WoLF PSORT (https://wolfpsort.hgc.jp/, accessed on 17 September 2021) and SignalP-5.0 (http://www.cbs.dtu.dk/services/SignalP/, accessed on 17 September 2021) servers were used to predict the subcellar locations and signal peptides of the PR-1 proteins. Alignment and visualization of the PR-1 domain were performed through DNAMAN 7.0 software.

### 4.3. Analysis of the Conserved Motifs, Gene Structures, and Protein Functional Networks

The conserved motifs were analyzed with the MEME platform (http://meme-suite.org/tools/meme, v4.9.0, accessed on 20 September 2021). The number of motifs was set to 20, and other parameters were set to default values [49]. The exon-intron structures were retrieved from the gene-annotation file (http://www.plantkingd omgdb.com/tea_tree/data/gff3/, accessed on 20 September 2021), and the diagrams were drawn by using TBtools v1.098661 software [50]. The functional-interaction networks of the PR-1 proteins in tea plants were analyzed based on the STRING protein-interaction database (http://string-db.org/, accessed on 21 September 2021).

### 4.4. Analysis of the Cis Elements of the CsPR-1 Gene Promoters

The *CsPR-1* promoter sequences (2 kb upstream the start codon) were retrieved from the TPIA (http://tpia.teaplant.org/index.html, accessed on 23 September 2021). PlantCARE online software (http://bioinformatics.psb.ugent.be/webtools/plantcare/html/, accessed on 23 September 2021) was used to search and analyze the cis elements in the extraction-promoter sequences. These elements were visualized using TBtool software [50].

### 4.5. Structural Analysis of CsPR-1 Proteins

Secondary structures were predicted with the SOPMA server (https://npsa-prabi.ibcp.fr/cgi-bin/npsa_automat.pl?page=/NPSA/npsa_sopma.html, accessed on 7 January 2022). Tertiary protein structures were predicted with the Phyre2 server (http://www.sbg.bio.ic.ac.uk/~phyre2/html/page.cgi?id=index, accessed on 7 January 2022). Disordered regions were predicted with the PONDR server (http://www.pondr.com/, accessed on 7 January 2022). The BetaCavityWeb server (http://voronoi.hanyang.ac.kr/betacavityweb, accessed on 7 January 2022) was used to search and analyze the channel structure. Web POCASA (http://g6altair.sci.hokudai.ac.jp/g6/service/pocasa/, accessed on 9 January 2022) was used to predict pocket structures.

### 4.6. PAS Staining

The samples were fixed with formaldehyde/acetic acid/ethanol fixative (containing 50% ethanol, 5% acetic acid, and 10% formaldehyde in H_2_O), dehydrated, transparented, waxed, and embedded. The paraffin sections were then routinely dewaxed in water and put into periodic acid solution for 10–15 min. Then, the slides were stained with PAS for 10 min. After being washed with water and dehydrated, the slices were covered with paraffin for further observation using the Olympus BX51 microscope (Olympus, Tokyo, Japan).

### 4.7. RNA Extraction and Quantitative RT-PCR Analysis

Total RNA was extracted using the Plant RNA Kit (Omega, Norcross, GA, USA). cDNA was synthesized by using the 5 × All-In-One RT MasterMix Kit (ABM, Richmond, BC, Canada) according to the manufacturer’s protocol, and the cDNA was diluted to 200 ng/μL for subsequent analysis. Then, qRT-PCR was performed using ChamQ SYBR qPCR Master Mix (Vazyme, Nanjing, China) on an iQ5 real-time PCR platform (Bio-Rad, Hercules, CA, USA) with the following PCR parameters: 95 °C for 30 s, followed by 40 cycles of 95 °C for 5 s and 60 °C for 30 s. Three independent biological replicates were performed, and the qPCR of each replicate was performed in triplicate. Relative transcript abundances of the *PR-1* genes were calculated via the 2^–ΔΔCT^ method [51]. All primers were designed using Primer5 software, and primer sequences are listed in Table S4.

## 5. Conclusions

**Figure 8:**
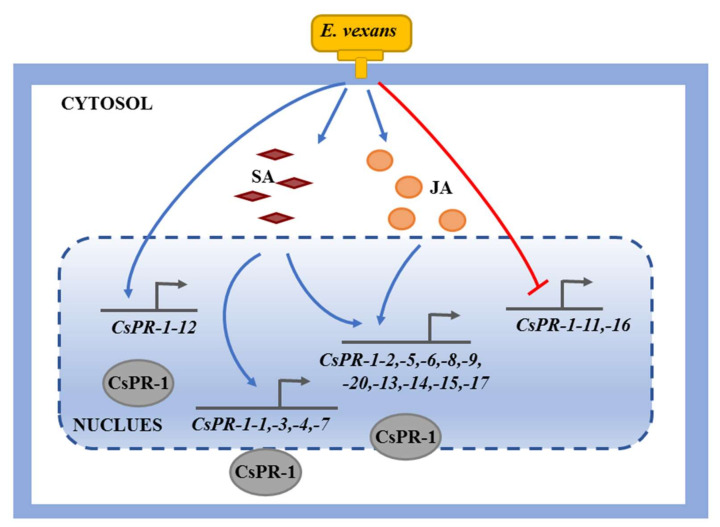
Proposed model of *CsPR-1*-modulated blister blight in tea plants. The blue arrow indicates the upregulation of genes; the red arrow indicates the downregulation of genes.

In the present study, 17 *CsPR-1* genes were identified in tea plants, and bioinformatics and expression-profile analyses were performed to determine their potential functions (Figure 8). The *CsPR-1* genes were found to be actively involved in the response to tea blister blight stress, and these processes are closely related to the signal transduction pathways involving TCA, NPR1, EDS16, BGL2, PR4, and HCHIB. The results provide new insights into the response to tea blister blight stress and also contribute to an important basis for subsequent functional studies investigating *CsPR-1* in tea plants.

## Figures and Tables

**Table 1:** Identified *PR1* genes in the genome of the tea plant.

Sequence ID	Gene	Chromosome	Protein					
			Length (aa)	MW (kD)	*p*I	Gravy	SP	Subcellular Location
TEA022692.1	*CsPR1-1*	Chr04	163	18.07	7.08	−0.353	24	extr: 7, chlo: 6, cyto: 1
TEA002521.1	*CsPR1-2*	Chr06	340	38.28	10.07	−1.089	27	vacu: 5, nucl: 3, cyto: 2, plas: 2, chlo: 1, E.R.: 1
TEA017943.1	*CsPR1-3*	Chr06	162	17.93	8.74	−0.213	24	extr: 6, vacu: 5, chlo: 2, golg: 1
TEA021774.1	*CsPR1-4*	Chr13	162	17.93	8.74	−0.213	24	extr: 6, vacu: 5, chlo: 2, golg: 1
TEA022146.1	*CsPR1-5*	Chr01	173	18.61	6.81	−0.227	21	extr: 4, chlo: 3, vacu: 3, cyto: 2, plas: 2
TEA004542.1	*CsPR1-6*	Chr06	160	17.59	4.96	−0.235	24	extr: 12, vacu: 2
TEA022585.1	*CsPR1-7*	Chr08	170	19.25	9.22	−0.375	24	chlo: 12, extr: 1, vacu: 1
TEA028234.1	*CsPR1-8*	Chr01	162	18.26	9.16	−0.399	24	chlo: 4, vacu: 3, extr: 2, E.R.: 2, nucl: 1, mito: 1, plas: 1
TEA004551.1	*CsPR1-9*	Chr06	159	17.52	8.47	−0.27	22	chlo: 14
TEA028218.1	*CsPR1-10*	Chr01	156	17.37	4.84	−0.406	28	chlo: 11, cyto: 2, nucl: 1
TEA022150.1	*CsPR1-11*	Chr01	191	21.53	9.16	−0.34	\	vacu: 6, chlo: 4, extr: 2, nucl: 1, plas: 1
TEA030748.1	*CsPR1-12*	unkown	204	22.60	5.66	−0.257	\	E.R.: 5.5, cyto: 5, E.R._plas: 3.5, nucl: 1, mito: 1, vacu: 1
TEA025681.1	*CsPR1-13*	Chr03	196	22.61	6.09	−0.479	23	extr: 7, chlo: 4, E.R.: 2, vacu: 1
TEA022240.1	*CsPR1-14*	unkown	162	17.88	6.86	−0.297	24	chlo: 13, vacu: 1
TEA021361.1	*CsPR1-15*	Chr03	160	17.62	5.38	−0.269	24	extr: 11, vacu: 2, cyto: 1
TEA011597.1	*CsPR1-16*	unkown	168	17.94	8.57	−0.213	24	chlo: 6, vacu: 5, extr: 2, golg: 1
TEA004541.1	*CsPR1-17*	Chr06	162	17.87	8.62	−0.345	24	extr: 7, vacu: 4, chlo: 2, mito: 1

**Table 2:** Structural analyses of the CsPR-1 proteins.

Proteins	α-Helices (%)	Extended Strand (%)	Beta Turn (%)	Random Coil (%)	Ramachandran Plot (%)	Number of Channels	Disordered Regions (%)
CsPR-1-1	38.65	13.50	4.29	43.56	98.3	2	23.93
CsPR-1-2	23.82	8.24	4.12	63.82	99.2	2	61.76
CsPR-1-3	37.04	14.20	6.17	42.59	98.3	4	24.07
CsPR-1-4	37.04	14.20	6.17	42.59	98.3	4	24.07
CsPR-1-5	30.64	17.34	1.73	50.29	96.0	3	20.23
CsPR-1-6	29.38	20.62	4.38	45.62	98.3	3	23.75
CsPR-1-7	29.41	17.65	4.71	48.24	97.4	3	6.47
CsPR-1-8	32.72	16.05	2.47	48.77	99.1	2	17.28
CsPR-1-9	33.33	15.09	3.77	47.80	99.1	3	18.24
CsPR-1-10	36.54	16.67	7.05	39.74	99.1	3	16.03
CsPR-1-11	39.27	15.18	4.71	40.84	96.7	5	9.42
CsPR-1-12	32.21	17.45	6.04	44.30	99.1	3	22.15
CsPR-1-13	28.57	22.45	3.06	45.92	96.7	9	16.33
CsPR-1-14	33.95	15.43	4.32	46.30	99.1	3	17.28
CsPR-1-15	38.12	16.25	4.38	41.25	98.3	3	27.50
CsPR-1-16	39.88	11.31	4.76	44.05	97.5	6	32.74
CsPR-1-17	37.04	16.05	4.32	42.59	99.1	3	18.52

## Data Availability

The data presented in this study are available within the article and Supplementary Material.

## Supplementary Materials

The following supporting information can be downloaded at: https://www.mdpi.com/article/10.3390/ijms23031292/s1.
