# Supplementary material for: Genome-Wide Characterization and Expression Analysis of Pathogenesis-Related 1 (PR-1) Gene Family in Tea Plant (Camellia sinensis (L.) O. Kuntze) in Response to Blister-Blight Disease Stress
Source: Int J Mol Sci. 2022 Jan 24;23(3):1292. doi: 10.3390/ijms23031292 (PMC8836084; doi:10.3390/ijms23031292)
Supplement: Supplementary file 1 [file ijms-23-01292-s001.zip › Table S2.pdf]

Table S2. Multilevel consensus motifs observed in CsPR-1 proteins

Motifs	E-value	Sequences
1	2.0e-318	ARAQVGVGPLTWBNTVAAYAQNYANLRSADCNLEHSNGPYG
2	1.2e-243	ENLAEGSGEFTGTDAVKLWVDEKPYYDYNSNSCVG
3	6.8e-195	CLHYTQVVWRNTARIGCARV
4	7.9e-136	FVTCNYDPPGNYIGZRPY
5	1.7e-118	LTMAHLSYAQNSPQDFLAAHN
6	4.1e-071	MALSKISLAIVSFMA
7	2.1e-012	CTNNGW
8	1.4e-007	HNDARAEVGVDPLKWSENLANGSSRLVRYQKNDMNCRFA
9	3.6e+000	MKPYFGJFFIFF
10	1.2e+002	KRQRNFFRCRD
11	1.1e+003	FGQYGENQLWLDYDEKPPEEVVZVWINEEK
12	1.5e+003	IGEMNQFLFPQN
13	2.1e+003	MAEIIL
14	2.8e+003	PRLWGKEREHRL
15	2.2e+003	WGTRLH
16	1.1e+003	PPPPNTT
17	4.6e+003	KHYKPC
18	4.7e+003	RINRGYRR
19	4.0e+003	DNQKCNC
