# Supplementary material for: Genome-Wide Characterization and Expression Analysis of Pathogenesis-Related 1 (PR-1) Gene Family in Tea Plant (Camellia sinensis (L.) O. Kuntze) in Response to Blister-Blight Disease Stress
Source: Int J Mol Sci. 2022 Jan 24;23(3):1292. doi: 10.3390/ijms23031292 (PMC8836084; doi:10.3390/ijms23031292)
Supplement: Supplementary file 1 [file ijms-23-01292-s001.zip › Table S4.pdf]

Table S4 Primers used for quantitative real-time PCR analysis

Target gene	Forward primer sequence (5′→3′)	Reverse primer sequence (5′→3′)
CsPR1-1	ATGGCTCTTTCCAAGATTTCAC	GTTCCATGCAAGAGGCTTGA
CsPR1-2	ATGGAAATGTCTAAGGCTCAAATCC	TTCCTGTGCTTCTGATGACAAC
CsPR1-3	ATGGCTCTCTCCAAGATTTCAC	GTTCCATGTAAGAGGCTTGACTC
CsPR1-4	ATGGCTCTCTCCAAGATTTCAC	GTTCCATGTAAGAGGCTTGACTC
CsPR1-5	ATGGCTGAGATCATACTAGCAC	CCCACTTCTGCTCTTGCTTGG
CsPR1-6	CTAGTAATTGTTTCCTTCATGGCC	ATAGTTTAGGGCATATGCCGC
CsPR1-7	AGCCACGGTATCATCACCATC	CCATGCCAATGGTTGCAT
CsPR1-8	CCATGCCAATGGTTGCAT	ATGGTGCTGTGTAAGCTTTCAT
CsPR1-9	ATGGTGCTGTGTAAGCTTTCAT	GTCCCATGTCATAGGTCCAA
CsPR1-10	GTCCCATGTCATAGGTCCAA	TGTGTGTCTCATAGCCTTGG
CsPR1-11	TGTGTGTCTCATAGCCTTGG	AATCGCCAATCCTTGAATTAGC
CsPR1-12	AATCGCCAATCCTTGAATTAGC	CCTTACAGGGAGAACCTTGC
CsPR1-13	CCTTACAGGGAGAACCTTGC	ACCCAAGATGGATGGACTTTC
CsPR1-14	ACCCAAGATGGATGGACTTTC	ATGTCCTACAAAACAGTCATAAGCC
CsPR1-15	ATGTCCTACAAAACAGTCATAAGCC	TTGTGTGCGTCTACGAACTGT
CsPR1-16	TTGTGTGCGTCTACGAACTGT	ATGGTGTGGGAAGATCTTGG
CsPR1-17	ATGGTGTGGGAAGATCTTGG	GCTAATGAAAGCTTACACAGCA
